# Paraxanthine provides greater improvement in cognitive function than caffeine after performing a 10-km run

**DOI:** 10.1080/15502783.2024.2352779

**Published:** 2024-05-09

**Authors:** Choongsung Yoo, Dante Xing, Drew E. Gonzalez, Victoria Jenkins, Kay Nottingham, Broderick Dickerson, Megan Leonard, Joungbo Ko, Megan H. Lewis, Mark Faries, Wesley Kephart, Martin Purpura, Ralf Jäger, Shawn D. Wells, Kylin Liao, Ryan Sowinski, Christopher J. Rasmussen, Richard B. Kreider

**Affiliations:** a Texas A&M University, Exercise & Sport Nutrition Laboratory, Human Clinical Research Facility, Department of Health & Kinesiology, College Station, TX, USA; b Texas A&M University, Texas A&M AgriLife Extension, College Station, TX, USA; c University of Wisconsin – Whitewater, Department of Kinesiology, Whitewater, WI, USA; d Increnovo LLC, Milwaukee, WI, USA; e Ingenious Ingredients LP, Lewisville, TX, USA

**Keywords:** Nootropic, caffeine alternative, ergogenic aid, sports nutrition

## Abstract

**Rationale:**

Intense exercise promotes fatigue and can impair cognitive function, particularly toward the end of competition when decision-making is often critical for success. For this reason, athletes often ingest caffeinated energy drinks prior to or during exercise to help them maintain focus, reaction time, and cognitive function during competition. However, caffeine habituation and genetic sensitivity to caffeine (CA) limit efficacy. Paraxanthine (PX) is a metabolite of caffeine reported to possess nootropic properties. This study examined whether ingestion of PX with and without CA affects pre- or post-exercise cognitive function.

**Methods:**

12 trained runners were randomly assigned to consume in a double-blind, randomized, and crossover manner 400 mg of a placebo (PL); 200 mg of PL + 200 mg of CA; 200 mg of PL + 200 mg of PX (ENFINITY®, Ingenious Ingredients); or 200 mg PX + 200 mg of CA (PX+CA) with a 7–14-day washout between treatments. Participants donated fasting blood samples and completed pre-supplementation (PRE) side effects questionnaires, the Berg-Wisconsin Card Sorting Test (BCST), and the Psychomotor Vigilance Task Test (PVTT). Participants then ingested the assigned treatment and rested for 60 minutes, repeated tests (PRE-EX), performed a 10-km run on a treadmill at a competition pace, and then repeated tests (POST-EX). Data were analyzed using General Linear Model (GLM) univariate analyses with repeated measures and percent changes from baseline with 95% confidence intervals.

**Results:**

BCST correct responses in the PX treatment increased from PRE-EX to POST-EX (6.8% [1.5, 12.1], *p* = 0.012). The error rate in the PL (23.5 [−2.8, 49.8] %, *p* = 0.078) and CA treatment (31.5 [5.2, 57.8] %, *p* = 0.02) increased from PRE-EX values with POST-EX errors tending to be lower with PX treatment compared to CA (−35.7 [−72.9, 1.4] %, *p* = 0.059). POST-EX perseverative errors with PAR rules were significantly lower with PX treatment than with CA (−26.9 [−50.5, −3.4] %, *p* = 0.026). Vigilance analysis revealed a significant interaction effect in Trial #2 mean reaction time values (*p* = 0.049, $\eta_{p}^{2}$ ^=^ 0.134, moderate to large effect) with POST-EX reaction times tending to be faster with PX and CA treatment. POST-EX mean reaction time of all trials with PX treatment was significantly faster than PL (−23.2 [−43.4, −2.4] %, *p* = 0.029) and PX+CA (−29.6 [−50.3, −8.80] %, *p* = 0.006) treatments. There was no evidence that PX ingestion adversely affected ratings of side effects associated with stimulant intake or clinical blood markers.

**Conclusions:**

Results provide some evidence that pre-exercise PX ingestion improves prefrontal cortex function, attenuates attentional decline, mitigates cognitive fatigue, and improves reaction time and vigilance. Adding CA to PX did not provide additional benefits. Therefore, PX ingestion may serve as a nootropic alternative to CA.

## 1. Introduction

Prolonged exercise promotes mental fatigue and can impair performance, particularly toward the end of competition when decision-making is often critical for success [1,2]. For this reason, athletes often consume energy drinks containing nootropic nutrients before and/or during exercise to help them maintain focus, cognitive function, and performance [3–8]. Caffeine is one of the most common naturally occurring nootropic nutrients in beverages that help maintain alertness, mental function, and exercise performance [7–9]. According to the International Society of Sports Nutrition, ingesting 3–6 mg/kg of caffeine about 60 minutes before exercise can improve cognition, attention, vigilance, and exercise performance [8,10]. However, the effects of ingesting caffeine before exercise vary depending on the type, amount, and length of exercise. Additionally, individuals with a homogenous A allele of the CYP1A2 gene tend to produce more cytochrome P450, an enzyme responsible for about 95% of caffeine metabolism and consequently metabolize caffeine more quickly [11]. Fast metabolizers of caffeine experience more significant ergogenic outcomes in some [11,12] but not all studies [13]. In addition, habituation to ingesting daily consumption of caffeinated foods and beverages can reduce its efficacy.

In humans, about 70% of CA is metabolized into 1,7-dimethylxanthine or paraxanthine (PX), with the remainder metabolized into 3,7-dimethylxanthine or theobromine (TB) and 1,3-dimethylxanthine or theophylline (TP) [14]. Compared to caffeine, paraxanthine has a shorter half-life and faster clearance from the blood than caffeine, theobromine, and theophylline [15]. Studies also indicate that paraxanthine has less toxic [16], anxiogenic [17], cardiovascular, and gastrointestinal side effects than caffeine [18]. Additionally, paraxanthine has a higher binding potency for adenosine A1 and A2a receptors and more substantial locomotor activation effects [19]. Paraxanthine has also been reported to inhibit phosphodiesterase 9 (PDE9), which terminates nitric oxide (NO) neurotransmission by metabolizing cyclic guanosine monophosphate (cGMP) back to GMP. Through PDE9 inhibition, paraxanthine potentiates NO neurotransmission, while caffeine does not affect this pathway [20]. Paraxanthine has been shown to have protective effects for dopaminergic neurons and has been reported to reduce synaptic function-related neurodegeneration, while caffeine provides marginal protection [21]. In addition, the wake-promoting potency of paraxanthine is greater and longer lasting than caffeine [22].

Theoretically, avoiding genetic and/or metabolically related variations in caffeine metabolism by supplementing directly with paraxanthine may provide a more direct way to improve cognitive and/or exercise performance with fewer side effects. In support of this hypothesis, we reported that ingestion of 200 mg of PX enhanced memory, reaction time, and attention for up to 6 hours in healthy adults [23]. Additionally, the ingestion of 50, 100, and 200 mg of PX for up to 7 days enhanced measures of cognition, memory, reasoning, response time, and helped sustain attention with no apparent side effects [24]. However, we are unaware of any study assessing the effects of ingesting paraxanthine prior to intense exercise on cognition following intense exercise. Moreover, we are not aware of any study that assessed the efficacy of paraxanthine supplementation compared to caffeine following exercise. Since staying focused and making quick decisions influences decision-making in sports and prolonged exercise promotes mental fatigue [2,25–27], we hypothesized that ingestion of paraxanthine prior to exercise may serve as an effective nootropic and/or ergogenic aid and potentially reducing exercise-induced mental fatigue. Therefore, the primary aims of this study were to determine: (1) whether acute paraxanthine ingestion affects cognitive function prior to and/or following exercise, (2) whether paraxanthine has measurable benefits in comparison to caffeine, and (3) whether the co-ingestion of paraxanthine and caffeine has additive or synergistic effects. If effective, acute paraxanthine ingestion could serve as a viable nootropic alternative to caffeine ingestion for energy drinks and/or pre-workout supplements designed to promote and/or sustain cognitive function during exercise.

## 2. Methods

### 2.1. Design of study

The experimental design was a randomized, crossover, double-blind, placebo-controlled clinical trial. The study was approved by the Human Protection Institutional Review Board (IRB2019–0928F) in accordance with ethical standards for the conduct of human participant research as described in the Declaration of Helsinki. This clinical trial was registered with the International Standard Randomized Control Number registry (ISRCTN14506218). Stimulant ingestion served as the independent. The primary outcome was measures of cognitive function. Secondary outcomes included changes in exercise heart rate, clinical blood chemistry panels, and subjective ratings of symptoms and side effects.

### 2.2. Study participants

**Figure 1.**
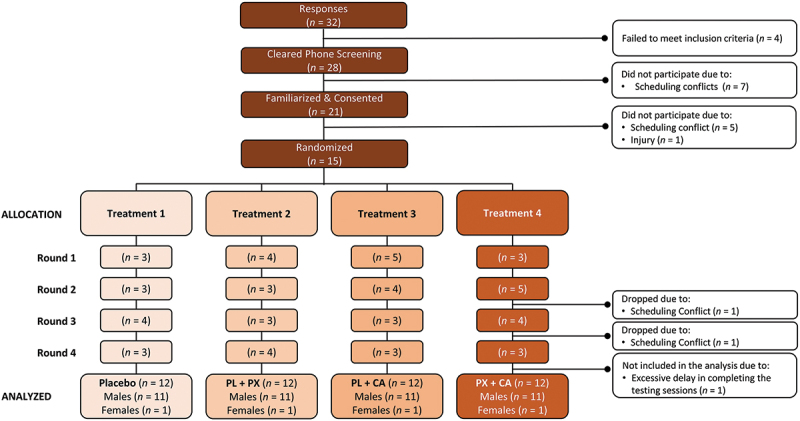
Consolidated standards of reporting trials (CONSORT) illustration for the placebo (PL), paraxanthine (PX), caffeine (CA), and paraxanthine + caffeine (PX+CA) treatments.

Trained runners from local running and triathlon clubs and races were recruited for this study. Eligibility criteria included healthy trained runners or triathletes between 18–40 years of age, current (≥6 months) history of run training, and documented evidence that they averaged 8 minutes/mile or less running pace during a recent competition (e.g. completing a 5-km road race or marathon). Qualified runners were invited to attend a familiarization session, which provided an overview of the study, and participants informed consent to participate in the study. Participants then completed a medical history and underwent a physical exam. Runners were not eligible to participate in the study if they had (1) a medical condition that hindered the ability to perform the study protocol; (2) a history of cognitive dysfunction; (3) were currently taking prescription medications; (4) a known allergy to wheat flour; (5) a sleep disorder; (6) been/were pregnant or breastfeeding; or (7) a physician’s order to abstain/restrict caffeine or stimulant intake. A Consolidated Standards of Reporting Trials (CONSORT) diagram is shown in Figure 1. A total of 32 potential participants responded to study advertisements and were assessed for eligibility. Of these, 28 passed the phone screening and were invited to familiarization sessions. Due to scheduling conflicts, 21 were familiarized and consented to participate in the study. Of these, 15 individuals were able to participate in the study and were randomized into testing sessions. Treatment allocaations are presented by testing rounds with the number (*n*) of participants tested shown. Due to pandemic-related delays in conducting human participant research, two participants moved during the study. One volunteer was omitted from the analysis because they could not complete testing sessions in a timely manner due to scheduling conflicts. Therefore, 12 runners (11 males, 1 female) were included in the analysis.

### 2.3. Testing protocol

Volunteers visited the lab five times, including one familiarization session and four experimental sessions. During the familiarization session, participants were explained the study protocol, provided informed consent, answered a medical questionnaire, and had height, weight, resting heart rate, and resting blood pressure. Males then had body composition determined using dual-energy X-ray absorptiometry (DXA), while females took a urine pregnancy test prior to the DXA scan to verify they were not pregnant. Participants then took each cognitive function test three times to familiarize themselves with the tests and establish test reliability. Volunteers practiced running on the treadmill to be used in the study at a competitive pace. Once completed, the participants performed a graded maximal cardiopulmonary (VO_2_ peak) treadmill test to determine peak heart rate and aerobic capacity. Participants then practiced the anaerobic capacity cycling test.

**Figure 2.**
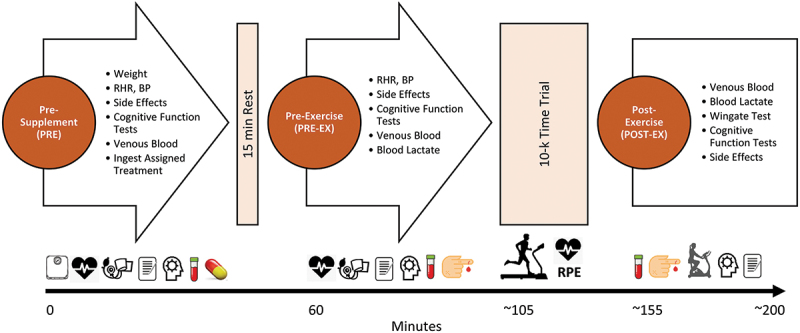
Overview of experiment study timeline. RHR represents resting heart rate, BP represents blood pressure.

Participants followed normal eating habits and abstained from ingesting new dietary supplements for the duration of the study. Participants prepared for each testing session as they would a 10-km road race. Additionally, they refrained from vigorous physical activity, alcohol intake, and over-the-counter medications for 24 hours as well as fasted for 8–12 hrs prior to reporting to the lab. Figure 2 shows the timeline of tests performed during each experimental testing session. Upon arriving at the lab, participants had their weight, resting heart rate, and blood pressure determined. Participants then completed a side effects questionnaire, performed cognitive function tests, donated a fasting blood sample, and then ingested 1 of 4 randomly assigned oral supplements (PRE). Participants then rested for 15 minutes and repeated these tests (PRE-EX). Volunteers then performed a 10-km run time trial at their self-determined pace. Performance times, heart rate, and rating of perceived exertion (RPE) were obtained every kilometer during the run. Once completed, participants donated a venous blood sample and then performed a 30-second anaerobic capacity test. Participants then completed the cognitive function tests and reported side effects (POST-EX). After each testing session, participants observed a 7 to 14-day washout period and then identically repeated the protocol while ingesting the remaining treatments in a randomized manner.

### 2.4. Supplementation protocol

A Balanced Latin Square method was used to counterbalance the order of treatments [28]. Treatments included (1) 400 mg of placebo (PL, wheat flour, Shandong Bailong Chuangyuan Bio-tec Co. Ltd., Dezhou, China); (2) 200 mg of PL +200 mg of CA (CSPC Innovation Pharmaceutical Co. Ltd., Shi Jiazhuang, China); (3) 200 mg of PL +200 mg of PX (ENFINITY®, Ingenious Ingredients, L.P. Lewisville, TX, USA) or (4) 200 mg CA +200 mg of PX (PX+CA). Supplements preparation followed good manufacturing practices and was certified by the manufacturer for content and purity. Supplements were similar in appearance and provided in generically labeled bottles. Supplements were administered after completing all PRE assessments on each testing day. Participants ingested one capsule with eight ounces of water of the assigned treatment, waited 15 minutes, and began post-supplementation assessments.

## 3. Procedures

### 3.1. Demographics

Weight and height were obtained using a Health-O-Meter Professional 500KL scale (Pelstar LLC, Alsip, IL, USA). Sitting resting heart rate and blood pressure were obtained after resting for 5-minutes. Resting heart rate was determined via palpation of the radial artery, while blood pressure was determined using a stethoscope and sphygmomanometer using standard procedures [29]. Body composition (excluding cranium) was determined using DXA (Hologic Inc., Waltham, MA, USA) with APEX Version 3.1 software (APEX Corporation Software, Pittsburg, PA, USA) [30,31] with test-retest and day-to-day variability found mean coefficients of variability (C_V_) for bone mineral content and lean mass of 0.31–0.45% with a mean intra-class correlation (ICC) of 0.985 [32,33]. Maximal aerobic capacity was determined from an incremental, symptom-limited, maximal cardiopulmonary exercise test using the Bruce treadmill protocol following standard procedures [34]. Aerobic capacity was determined using a ParvoMedics TrueMax 2400 Metabolic Measurement System (ParvoMedics Inc, Sandy, UT). The system volume measurement was calibrated with a series 5530 three-liter volume syringe (Hans Rudolph Inc., Kansas City, MO). Oxygen and carbon dioxide analyzers were calibrated to known medical-grade gases prior to each test following standard procedures.

### 3.2. Diet control

Participants prepare for testing sessions as they would leading up to a road race. For diet consistency, participants recorded food and beverage intake for 4 days prior to each testing session using the MyFitnessPal Calorie Counter phone application (MyFitnessPal, Inc., Baltimore, MD, USA) or written food logs [35,36]. Participants were asked to maintain caffeine intake (e.g. <200 mg/d) and refrain from ingesting any other stimulants not commonly consumed in their diet for 48 hours prior to each testing session as well as reported to the lab after an 8–12 hour fast to normalize diet and caffeine intake on performance. Participants replicated this diet prior to each testing session. Diet inventories were reviewed for consistency by one research assistant and analyzed using standard nutritional analysis software (Food Processor Version 11.4.412, ESHA Nutrition Research, Salem, OR, USA) [37].

### 3.3. Running performance assessment

The 10-km run was performed on a TrackMaster tmx425c treadmill (Full Vision INC., Newton, KS). Participants warmed up as they were accustomed to before competitive running and were then asked to perform each run to the best of their ability. Split times and RPE using the Borg 6–20 scale [38] were recorded at 1 km intervals. Heart rate was monitored using a Polar H10 Heart Rate Monitor (Polar Electro, Inc., Bethpage, NY, USA). Participants were offered water ad libitum at 1 km intervals. The volume from the first trial was used as a standard for the remaining trials. After completing the 10-km run and donating a blood sample for lactate determination, participants performed a 30-sec Wingate anaerobic cycling test on a Lode Excalibur Sport 925,900 cycle ergometer (Lode BV, Groningen, The Netherlands) at a standardized work rate of 7.5 J/kg/rev. Test-to-test variability of repeated Wingate anaerobic capacity tests in our laboratory yielded correlation coefficients of *r* = 0.98 ± 15% for mean power.

### 3.4. Cognitive assessment

Cognitive function was assessed by having participants perform the Psychology Experiment Building Language (PEBL) test (Version 2.1, http://pebl.sourceforge.net, accessed 19 June 2019) [39]. A more complete description of the PEBL tests employed in our lab were previously described [23]. The assessment battery included the Berg-Wisconsin Card Sorting Task test (BCST) that assesses reaction time and accuracy of sorting cards and thereby assesses reasoning, learning, executive function, attention shifting (i.e. flexibility in responding to changing schedules of reinforcement), and impulsiveness [39–43]; and, the Psychomotor Vigilance Task Test (PVTT) that assesses sustained attention and reaction times by pressing a key on a keyboard when a randomly illuminating light is displayed on a monitor every few seconds [40,41,44–46]. Participants practiced the test sessions during the familiarization session three times to establish test reliability. During each testing session, tests were administered in the same order and took about 30–35 minutes to complete. Participants were allowed to relax between each cognitive function test with no more than 5 minutes between trials.

### 3.5. Blood analysis

Fasting blood was obtained before treatment ingestion, before performing the run, and after the run. This involved taking about 10 mL of blood from an antecubital vein in the forearm using standard phlebotomy procedures [47,48]. Blood was collected in serum separation (SST) and K2 EDTA BD Vacutainer® tubes (Becton, Dickinson and Company, Franklin Lakes, NJ, USA). The SST tubes were left at room temperature for 15 minutes and then centrifuged for 10 minutes at 3,500 g in a refrigerated (4°C) Thermo Scientific Heraeus MegaFuge 40 R Centrifuge (Thermo Electron North America LLC, West Palm Beach, FL, USA) [49]. Clinical Pathology Labs, Inc. (Austin, TX. CLIA #45D0505003, CAP Accreditation #21525–01) analyzed whole blood and serum samples. Whole blood cell counts were analyzed by an automated multichannel hematology analyzer. A Roche Cobas Gen 2 analyzer (Roche Diagnostics International AG, Rotkreuz, Switzerland) was used to assess serum samples. Test-retest reliability of performing the assays evaluated in this lab ranged from 2% to 6%. Additionally, prior to and following the performance runs, about 0.7 µL of arterialized venous blood was obtained from a clean finger and measured for blood lactate using a calibrated Lactate Plus Meter (Nova Biomedical, Waltham, MA). Intra-analyzer reliability of the device demonstrated a typical error of measurement of 0.4 mM, with C_V_ values at 8.5% [50].

### 3.6. Side effect questionnaire

The frequency and severity of dizziness, tachycardia, heart palpitations, shortness of breath, blurred vision, and nervousness were assessed using a Likert-type scale where 0 represented none; 1 represented 1–2 per week or minimal; 2 represented 3–4 per week or slight; 3 represented 5–6 per week or moderate; 4 represented 7–8 per week or severe; and 5 represented ≥ 9 per week or very severe. Volunteers were also asked to report any other side effects they may have experienced in response to taking the assigned treatments. Reliability in answering these side effects questions in our lab revealed mean CVs ranging from 1.2–2.6% with a mean ICC ranging between 0.59–0.88 [51].

### 3.7. Statistical analysis

Data were analyzed by the IBM® Version 29 SPSS® statistical analysis software (IBM Corp., Armonk, NY, USA). The sample size was selected based on our previous work in this area [23,52–54] assuming an expected improvement of 5% with a power of 80% in primary outcome cognitive function-related variables. We previously demonstrated that the sample size was sufficient to assess clinically significant results [52–56]. A balanced Latin Square designer program [28] was used to randomize participants to treatments in a crossover manner. General linear model (GLM) multivariate and univariate analyses with repeated measures were used to analyze the data. The Wilks’ Lambda and Greenhouse-Geisser univariate correction tests assessed Time and Treatment x Time interaction effects. The probability of type I error was *p* < 0.05. Statistical tendencies were noted when *p*-values >0.05 to < 0.10 were observed. Pairwise comparisons were assessed using Fisher’s least significant difference statistics. We also analyzed data using relative dose as a covariate. Since the results were similar, we did not report this analysis. Mean changes from baseline with 95% confidence intervals (CIs) were used to assess the clinical significance of findings. Means and 95% CI’s completely above or below baseline were considered clinically significant [57]. Data are presented means and ± standard deviations (SD) or mean changes from baseline with lower and upper confidence intervals (mean [LL, UL]). Partial Eta squared ($\eta_{p}^{2}$) values were used to assess effect size where values of 0.01 represented a small effect, 0.06 represented a medium effect, and 0.14 represented a large effect size [58]. This statistical approach is consistent with recommendations from Earnest et al. [59] on best practices in reporting sport nutrition-related research.

## 4. Results

### 4.1. Demographic data

Table S1 shows participant demographic data. Twelve trained runners completed this study (11 males and 1 female). Participants were 26.4 ± 5.1 years old, 1.76 ± 0.1 m tall, weighed 68.6 ± 9.6 kg, had a body mass index (BMI) of 22.2 ± 2.8 kg/m^2^, 16.2 ± 5.2 percent body fat, had a peak oxygen uptake of 52.4 ± 10.6 ml/kg/min (15.0 ± 3.0 metabolic equivalents or METS), and peak heart rate observed of 185 ± 9.9 bpm. Participants also had a resting heart rate of 59.6 ± 10.7 bpm, a systolic blood pressure of 115.4 ± 8.3 mmHg, and a diastolic blood pressure of 68.9 ± 6.1 mmHg.

### 4.2. Performance analysis

Table S2 in the Supplemental Materials file presents split times, heart rate, RPE, and pre-and post-run blood lactate values observed during the 10 km runs. An overall GLM time effect was observed among split times, heart rate, and RPE (*p* < 0.001, $\eta_{p}^{2}$ = 0.902, large effect) with no significant treatment by interaction effects observed (*p* = 1.000, $\eta_{p}^{2}$ = 0.025, small effect). Comparable results were observed in univariate analyses. Blood lactate increased from pre- to post-exercise by 5.34 [4.57, 6.10] mmol/L, *p* < 0.001). However, no significant differences were observed among treatments (*p* = 0.738, $\eta_{p}^{2}$ = 0.028, small effect). Participants completed the runs in an average of 48.37 ± 6.75 min with an average heart rate of 185.7 ± 11.4 bpm, an RPE of 18.4 ± 1.7 on the 6–20 scale, and a blood lactate of 7.38 ± 2.5 mmol/L. No significant differences were observed between treatments with 1 km data. Similarly, no significant differences were observed among treatments in post-run anaerobic capacity (Table S3).

### 4.3. Cognitive function assessment

#### 4.3.1. Berg-Wisconsin card sorting test

**Figure 3.**
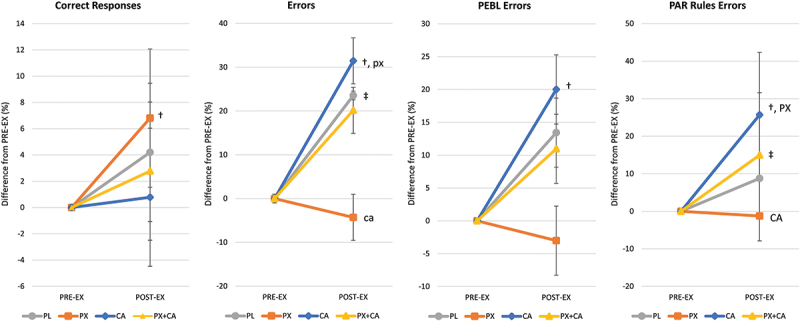
Berg-Wisconsin Card Sorting test mean percent changes from pre-exercise (PRE-EX) values with 95% confidence intervals. † represents *p* < 0.05 from PRE-EX values while ‡ represents *p* > 0.05 to *p* < 0.10 effect. Treatment differences (*p* < 0.05) are shown as differences from placebo (PL), paraxanthine (PX), caffeine (CA) and PX + CA. Statistical trends (*p* > 0.05 to *p* < 0.10) are shown as small case (pl, px, ca, px+ca).

Table S4 in the Supplemental Materials file presents the BCST results observed, while Figure S1a in the Supplemental Materials file presents individual and mean responses observed. Analysis of BCST data results revealed no significant multivariate or univariate treatment x time interaction effects in correct responses, errors, PEBL calculated perseverative errors, or perseverative errors with PAR rules (revised scoring method). Correct responses increased from PRE to POST-EX with PL treatment (*p* = 0.011) and from PRE-EX to POST-EX in the PX treatment (*p* = 0.028). A moderate treatment x time effect size was seen in errors ($\eta_{p}^{2}$ = 0.072, medium effect) with pairwise comparison analysis indicating that PRE-EX values between the PX and PX+CA treatments tended to differ (3.30 [−0.4, 6.6], *p* = 0.077). No significant time or treatment x time effects were seen in PEBL perseverative errors. However, a moderate effect size was observed in perseverative errors with PAR rules ($\eta_{p}^{2}$ = 0.077, medium effect). Treatment with CA increased preservative errors with PAR rules from PRE-EX to POST-EX (*p* = 0.032). Analysis of percent changes from PRE-EX to POST-EX is presented in Figure 3 below, while Figure S1b (shown in the Supplemental Materials file) shows mean changes from baseline with individual data points. Results revealed that correct responses in the PX treatment increased from PRE to POST-EX (6.8 [1.5, 12.1] %, *p* = 0.012). Errors in the PL (23.5 [−2.8, 49.8] %, *p* = 0.078) and CA treatment (31.5 [5.2, 57.8] %, *p* = 0.02) increased from PRE-EX values with POST-EX errors tending to be lower with PX treatment compared to CA (−35.7 [−72.9, 1.4] %, *p* = 0.059). Perseverative errors increased from PRE-EX to POST-EX values with CA treatment (20.0 [2.6, 37.4] %, *p* = 0.025). Finally, perseverative errors with PAR rules increased from PRE-EX to POST-EX with CA treatment (25.7% [9.0, 42.3], *p* = 0.003) with POST-EX values with PX treatment significantly lower than CA (−26.9% [−50.5, −3.4], *p* = 0.026). These findings provide evidence that PX ingestion significantly improves correct responses over time while significantly reducing errors in comparison to CA ingestion. However, PX treatment did not improve BCST performance measures compared to PL treatment.

#### 4.3.2. Psychomotor vigilance task test

**Figure 4.**
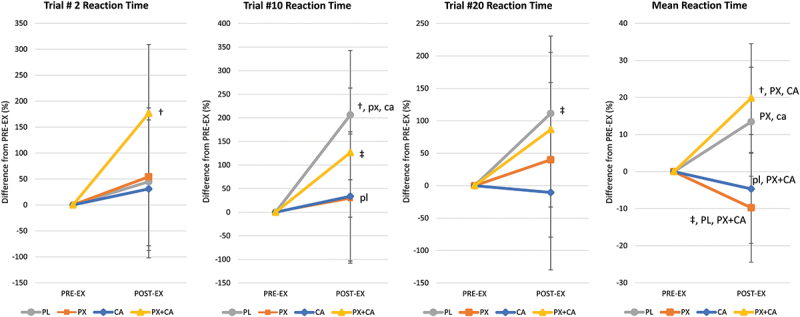
Psychomotor vigilance task mean percent changes from PRE-EX values with 95% confidence interval data. Mean reaction time is the mean for all 10 trials performed. † represents *p* < 0.05 from PRE-EX values while ‡ represents *p* > 0.05 to *p* < 0.10 trend. Treatment differences (*p* < 0.05) are shown as differences from placebo (PL), paraxanthine (PX), caffeine (CA) and PX + CA. Statistical trends (*p* > 0.05 to *p* < 0.10) are shown as small case (pl, px, ca, px+ca).

Table S3 in the Supplemental Materials file shows PVTT-related variables while Figure S2a in the Supplemental Materials file presents individual and mean responses observed. A significant univariate treatment x time effect with a large effect size was observed in trial #2 mean reaction time values (*p* = 0.049, $\eta_{p}^{2}$ ^=^ 0.134, medium to large effect). Pairwise comparisons revealed that reaction time increased with PL from PRE to POST-EX (1057 [365, 1750] ms, *p* = 0.004) while CA values tended to increase over time (618 [−74, 1310] ms, *p* = 0.079) in Trial #2. Post-EX trial #2 reaction times tended to be faster with PX and CA compared to PX+CA treatment. There was also evidence that mean reaction time increased over time with PL and PX+CA treatment while tending to decrease from PRE-EX to POST-EX with PX ingestion. Analysis of mean changes from PRE-EX values with 95% CI’s (Figure 4) revealed that reaction times increased in the PL and PX+CA treatments while PX and CA ingestion promoted faster reaction times to perform the PVTT. This was more evident when comparing POST-EX mean reaction times for all twenty trials. POST-EX mean reaction times in the PX treatment were significantly faster than PL (−23.2 [−43.4, −2.4] %, *p* = 0.029) and PX + CA (−29.6 [−50.3, −8.80] %, *p* = 0.006) while not significantly different than CA (−5.1 [−25.5, 15.7] %, *p* = 0.626). However, PX + CA ingestion promoted a greater improvement in mean reaction times compared to CA (−24.5 [−45.3, −3.7] %, *p* = 0.022). Figure S2b in the Supplemental Materials file shows the mean changes from baseline with individual data points. Results provide evidence that PX supplementation increases early and overall attention and prevents exercise-induced attention lapses compared to PL and in a similar manner as CA. However, ingesting PX+CA did not provide additive benefits.

### 4.4. Safety assessment

Tables S6 - S9 in the Supplemental Materials file show blood-related data. No significant multivariate or univariate interaction effects were observed in whole blood cell counts (Table S6), blood lipids (Table S7), markers of liver function (Table S8), and most markers of renal function (Table S9). Significant differences were observed among treatments in serum potassium and carbon dioxide, with PRE-EX and POST-EX values in the CA and PX+CA lower than PL values. Observed changes between PRE-EX and POST-EX were deemed exercise-induced and unrelated to supplementation. Tables S10 and S11 in the Supplemental Materials present the frequency and severity of common side effects associated with taking supplements, respectively. Results revealed no significant interactions among treatments. Several pairwise differences were observed among treatments, particularly in POST-EX responses. However, the mean rating values were typically less than 1.0, indicating that the side effects were infrequent and of minimal severity and associated with CA ingestion. There was no evidence that PX alone promoted any side effects. These findings suggest that acute ingestion of PX was well tolerated.

## 5. Discussion

Athletes often consume caffeinated beverages to increase energy, maintain alertness and/or provide cognitive or ergogenic benefits [1,4,9,10,60,61]. While caffeine’s pharmacokinetics and ergogenic value have been well documented [7,8,15,60,62–64], less is known about paraxanthine. We recently reported evidence that acute ingestion of 200 mg of paraxanthine ingestion influenced memory, cognition, and attention in healthy male and female participants [54]. A dose-response study confirmed the nootropic effects of paraxanthine in acute doses as little as 50 mg [24]. Moreover, Jäger and coworkers [65] reported that paraxanthine supplementation increased muscle mass, strength, and endurance in mice. While preclinical and mechanistic data suggest some advantages of paraxanthine over caffeine, no study has compared the nootropic effects of ingesting paraxanthine with or without caffeine on cognition after intense exercise. Demonstrating that paraxanthine supplementation has independent and/or synergistic effects on cognition after intense exercise compared to caffeine could provide evidence to support use of paraxanthine as an alternative to caffeine in pre-workout supplements and energy drinks.

Present findings add to initial findings that acute paraxanthine has nootropic properties. In this regard, analysis of the Berg Card Sorting Test results revealed that correct responses increased from PRE to POST-EX by 6.8% (*p* = 0.012) with paraxanthine ingestion, while POST-EX errors with paraxanthine ingestion tended to be lower than CA (−35.7%, *p* = 0.059). POST-EX perseverative errors with PAR rules were also significantly lower with PX treatment compared to CA (−26.9%, *p* = 0.026) while not significantly different than PL (−10.0 [−33.6, 13.5] %, *p* = 0.40). The BCST assesses thought, reasoning, learning, executive control, attention shifting, and impulsiveness [42,43]. Increases in perseverative errors are indicative of greater mental fatigue [66]. These findings provide evidence that paraxanthine ingestion can help sustain attention and improve accuracy over time. However, it should be noted that PX treatment did not improve BCST performance measures compared to PL treatment. Participants also performed the PVTT that assesses sustained attention reaction times to visual stimuli [44–46]. Our previous studies [54,56] reported that paraxanthine helped sustain attention over time. In the current study, paraxanthine ingestion prior to exercise improved reaction times during trial #2 of the PVTT while promoting faster POST-EX reaction times than observed after placebo ingestion PL (−23.2 [−43.4, −2.4] %, *p* = 0.029) and PX+CA (−29.6 [−50.3, −8.80] %, *p* = 0.006) while not significantly different than CA (−5.1 [−25.5, 15.7] %, *p* = 0.626). Nitric oxide neurotransmission plays a vital role in both the learning process and memory of the learned task [67]. Paraxanthine inhibits PDE9 and thereby potentiates NO neurotransmission, while caffeine does not have that effect. In addition, paraxanthine releases neurotransmitters (e.g. dopamine and glutamine) to a greater extent than caffeine. Although we did not assess neurotransmitter levels in the current study, the differences in neurotransmission between paraxanthine and caffeine treatments could explain measurable differences observed in cognitive performance (i.e. faster responses with fewer mistakes). The BCST was used to assess learning, and in our study, paraxanthine ingestion significantly reduced total errors POST-EX in comparison to caffeine (−35.8% (−72.9, 1.4), *p* = 0.059) and PAR rule errors (−26.9 (−50.5, −3.4), *p* = 0.026) while significantly increasing the number of correct responses from PRE-EX to POST-EX (6.8% (1.5, 12.1), *p* = 0.012). These findings provide evidence that acute ingestion paraxanthine significantly improves correct responses over time while significantly reducing errors compared to caffeine ingestion.

The Psychomotor Vigilance Task Test assesses sustained attention and reaction times over time by performing twenty test trials. Delays in response are considered lapses in attention. We previously reported that acute ingestion of paraxanthine sustained attention over time. In the present study, we found that paraxanthine ingestion before exercise promoted faster overall mean response times than the placebo (−23.2 [−43.4, −2.4] %, *p* = 0.029) and paraxanthine + caffeine treatment (−29.6 [−50.3, −8.80] %, *p* = 0.006). while not significantly different than CA (−5.1 [−25.5, 15.7] %, *p* = 0.626). However, paraxanthine + caffeine ingestion promoted a greater improvement in mean reaction times compared to caffeine alone (−24.5 [−45.3, −3.7] %, *p* = 0.022). Results provide some evidence that paraxanthine supplementation increased early and overall attention and prevents exercise-induced attention lapses compared to ingesting a placebo and in an analogous manner as caffeine. Interestingly, co-ingesting paraxanthine and caffeine did not provide additive benefits. These findings suggest that paraxanthine may have independent nootropic effects than caffeine.

Participants in our initial studies did not report any side effects from paraxanthine supplementation [23,24]. Conversely, caffeine and/or other stimulants have been reported to experience unwanted side effects in some individuals [60,68]. In the current study, caffeine intake increased perceptions of the frequency of tachycardia and shortness of breath over time while increasing perceptions of the severity of tachycardia, shortness of breath, and nervousness. Conversely, paraxanthine ingestion did not affect perceptions of the frequency or severity of these side effects. In fact, perceptions of the frequency of nervousness were significantly lower after exercise. Additionally, the paraxanthine treatment was associated with lower frequency and severity of monitored side effects than when ingesting caffeine or PX+CA before exercise. These findings support contentions that paraxanthine may be better tolerated than caffeine while still providing nootropic benefits [15,22].

A strength of this investigation is that it represents the first double-blind, placebo-controlled, crossover study in healthy younger individuals that directly compared the effects of pre-exercise paraxanthine ingestion with and without caffeine on cognitive function and psychomotor vigilance. Results add to accumulating evidence that paraxanthine may serve as a safe and effective nootropic nutrient. However, the study is limited by sample size, particularly in terms of the number of women studied. Additionally, although we asked participants to refrain from excessive stimulant intake during the study, differences in habitual diets and stimulant sensitivity to caffeine and/or paraxanthine may have varied, thereby influencing results. Further, although participants practiced the cognitive tests, natural day-to-day variability in cognitive function, mood, and/or motivation to perform cognitive function tests may have influenced the results. Further research is warranted to corroborate findings and explore whether individual variability, sensitivity in response to acute paraxanthine ingestion, caffeine restriction prior to use of paraxanthine, sex differences, and/or length of supplementation protocols may affect responsiveness to paraxanthine ingestion for cognitive and psychomotor enhancement.

## 6. Conclusion

Acute paraxanthine ingestion is safe and improved some measures of executive function, attenuate attentional degradation, and mitigate cognitive fatigue before and after exercise. We also observed some evidence that paraxanthine ingestion promoted more significant improvements than caffeine independently while co-ingesting paraxanthine with caffeine did not provide any additional benefit. These findings indicate that paraxanthine may serve as a viable alternative to caffeine in helping maintain cognitive function during prolonged exercise.

## Supplementary Material

Supplemental Material

## Data Availability

Data and statistical analyses are available upon request on a case-by-case basis for noncommercial scientific inquiry and/or educational use if IRB restrictions and research agreement terms are not violated.
