# Supplementary material for: Paraxanthine provides greater improvement in cognitive function than caffeine after performing a 10-km run
Source: J Int Soc Sports Nutr. 2024 May 9;21(1):2352779. doi: 10.1080/15502783.2024.2352779 (PMC11089923; doi:10.1080/15502783.2024.2352779)
Supplement: Supplemental Material [file RSSN_A_2352779_SM8199.docx]

| 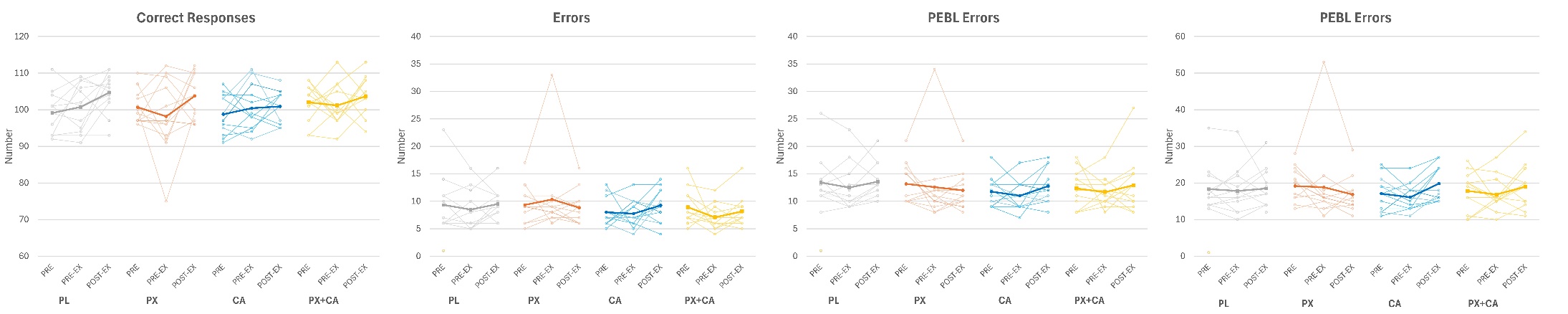  **Figure S1a:** Individual Berg-Wisconsin Card Sorting Test results. Data are from pre-supplementation (PRE), 60-min post-supplementation (PRE-EX) and post-exercise (POST-EX) data points with means and 95% confidence intervals for the placebo (PL), paraxanthine (PX), caffeine (CA) and PX + CA treatments.  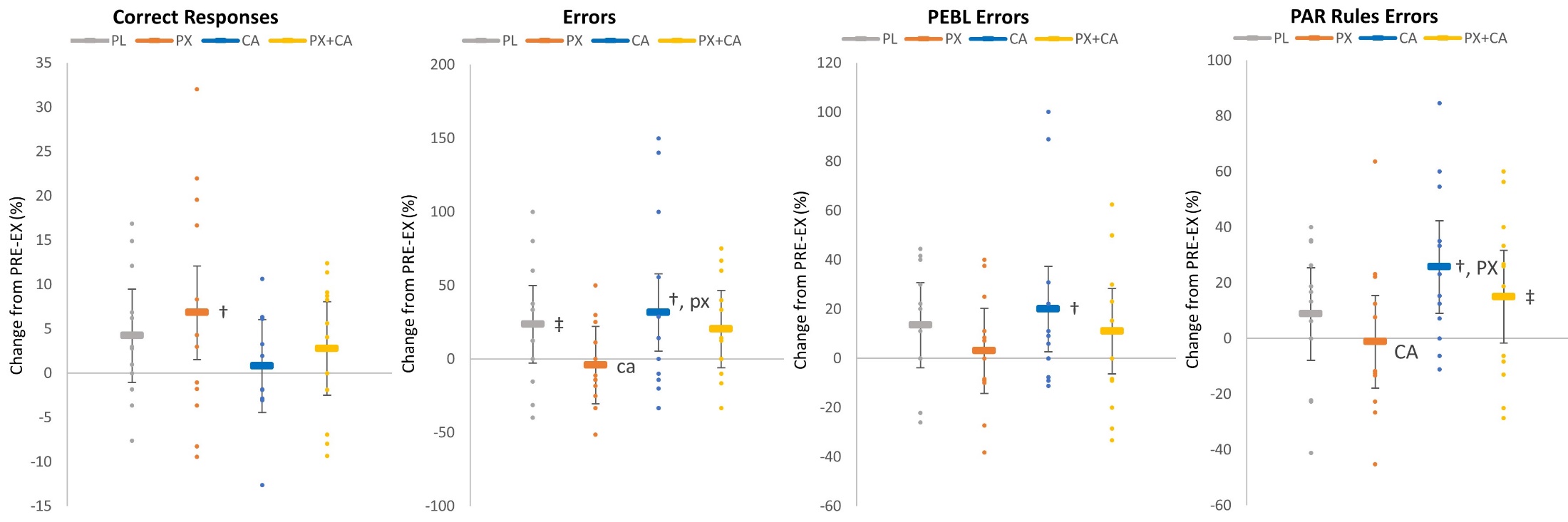  **Figure S1b:** Berg-Wisconsin Card Sorting Test mean percent change data. Data are from pre-exercise (PRE-EX) values with 95% confidence intervals. † represents p < 0.05 from PRE-EX values while ‡ represents p > 0.05 to p < 0.10 effect. Treatment differences (p < 0.05) are shown as differences from placebo (PL), paraxanthine (PX), caffeine (CA) and PX + CA. Statistical trends (p > 0.05 to p < 0.10) are shown as small case (pl, px, ca, px+ca). |  |
| --- | --- |
|  |  |


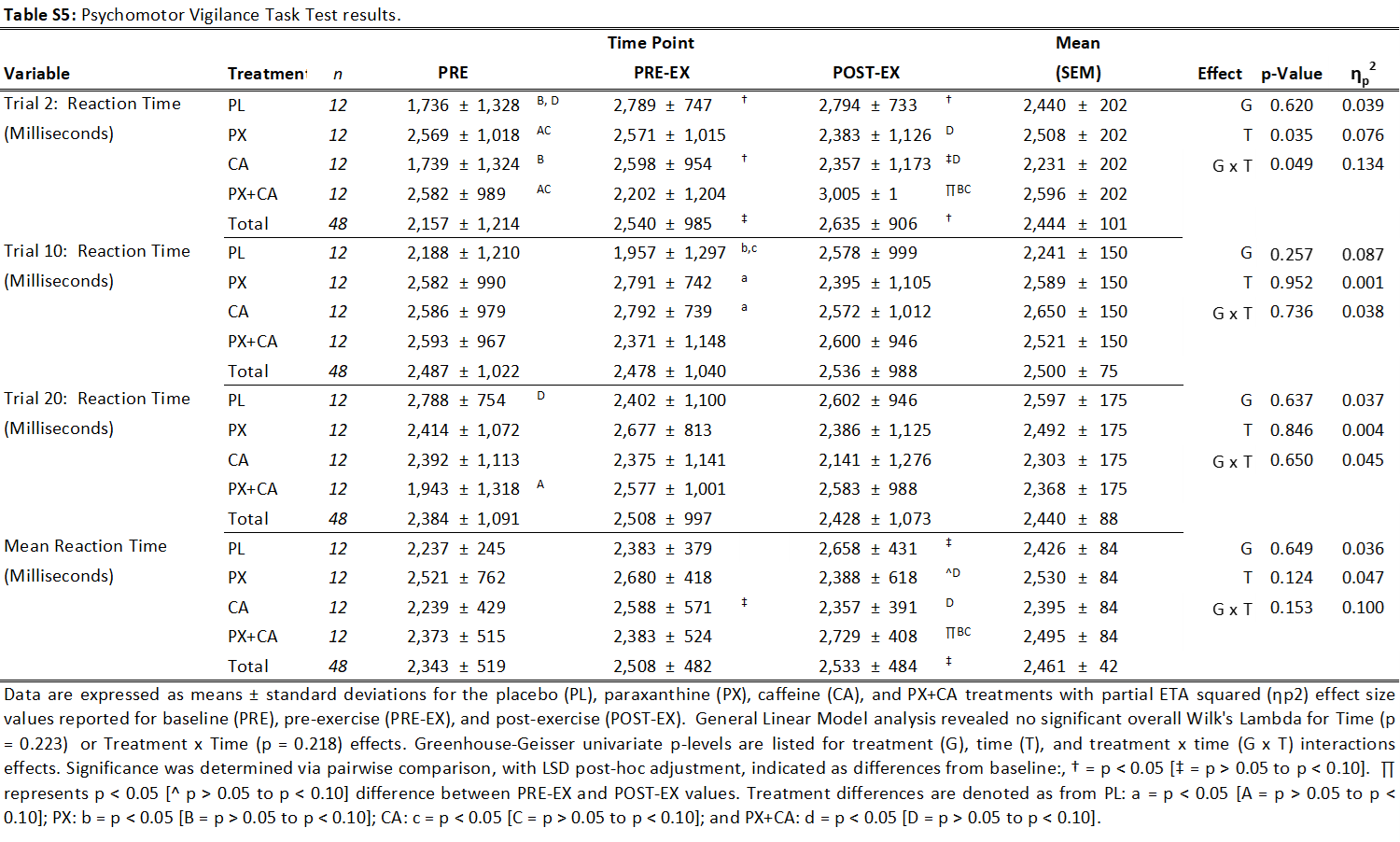


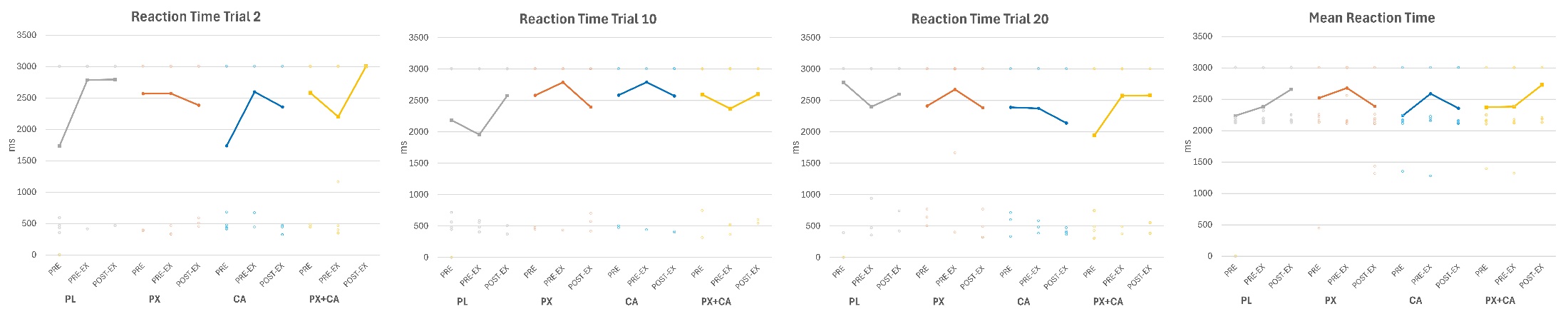


**Figure S2a.** Individual Psychomotor Vigilance Task results with individual data points. Data are from pre-supplementation (PRE), 60-min post-supplementation (PRE-EX) and post-exercise (POST-EX) data points with means and 95% confidence intervals for the placebo (PL), paraxanthine (PX), caffeine (CA) and PX + CA treatments.


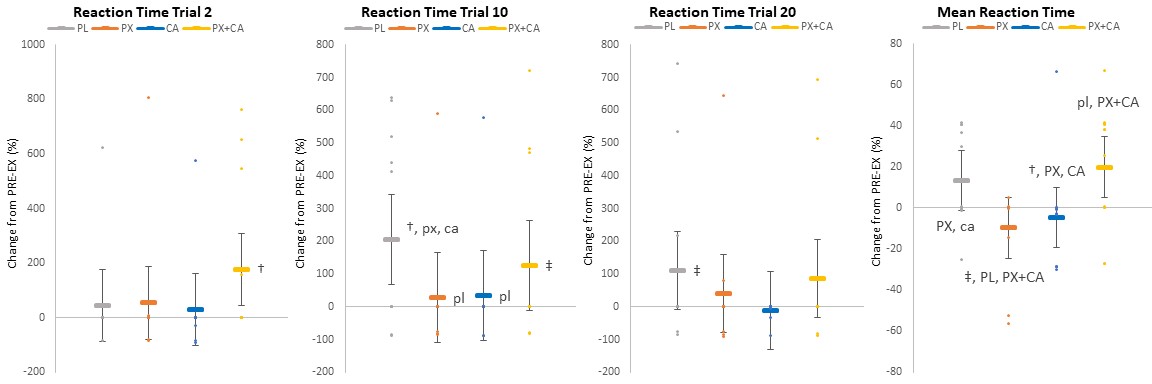


**Figure S2b.** Psychomotor Vigilance Task mean percent changes with individual data points. Data are from PRE-EX values with individual data points and means with 95% confidence interval data shown. Mean reaction time is the mean for all 10 trials performed. † represents p < 0.05 from PRE-EX values while ‡ represents p > 0.05 to p < 0.10 trend. Treatment differences (p < 0.05) are shown as differences from placebo (PL), paraxanthine (PX), caffeine (CA) and PX + CA. Statistical trends (p > 0.05 to p < 0.10) are shown as small case (pl, px, ca, px+ca).
